# As Time Goes by: Understanding Child and Family Factors Shaping Behavioral Outcomes After Traumatic Brain Injury

**DOI:** 10.3389/fneur.2021.687740

**Published:** 2021-07-05

**Authors:** Linda Ewing-Cobbs, Janelle J. Montroy, Amy E. Clark, Richard Holubkov, Charles S. Cox, Heather T. Keenan

**Affiliations:** ^1^Department of Pediatrics and Children's Learning Institute, McGovern Medical School, University of Texas Health Science Center at Houston, Houston, TX, United States; ^2^Department of Pediatrics, Division of Critical Care, School of Medicine, University of Utah, Salt Lake City, UT, United States; ^3^Department of Pediatric Surgery, McGovern Medical School, University of Texas Health Science Center at Houston, Houston, TX, United States

**Keywords:** traumatic brain injury, behavioral symptoms, psychological adjustment, emotional symptoms, conduct problems, executive functions, long-term outcome, pediatric

## Abstract

**Objective:** To model pre-injury child and family factors associated with the trajectory of internalizing and externalizing behavior problems across the first 3 years in children with pediatric traumatic brain injury (TBI) relative to children with orthopedic injuries (OI). Parent-reported emotional symptoms and conduct problems were expected to have unique and shared predictors. We hypothesized that TBI, female sex, greater pre-injury executive dysfunction, adjustment problems, lower income, and family dysfunction would be associated with less favorable outcomes.

**Methods:** In a prospective longitudinal cohort study, we examined the level of behavior problems at 12 months after injury and rate of change from pre-injury to 12 months and from 12 to 36 months in children ages 4–15 years with mild to severe TBI relative to children with OI. A structural equation model framework incorporated injury characteristics, child demographic variables, as well as pre-injury child reserve and family attributes. Internalizing and externalizing behavior problems were indexed using the parent-rated Emotional Symptoms and Conduct Problems scales from the Strengths and Difficulties questionnaire.

**Results:** The analysis cohort of 534 children [64% boys, *M* (SD) 8.8 (4.3) years of age] included 395 with mild to severe TBI and 139 with OI. Behavior ratings were higher after TBI than OI but did not differ by TBI severity. TBI, higher pre-injury executive dysfunction, and lower income predicted the level and trajectory of both Emotional Symptoms and Conduct Problems at 12 months. Female sex and poorer family functioning were vulnerability factors associated with greater increase and change in Emotional Symptoms by 12 months after injury; unique predictors of Conduct Problems included younger age and prior emotional/behavioral problems. Across the long-term follow-up from 12 to 36 months, Emotional Symptoms increased significantly and Conduct Problems stabilized. TBI was not a significant predictor of change during the chronic stage of recovery.

**Conclusions:** After TBI, Emotional Symptoms and Conduct Problem scores were elevated, had different trajectories of change, increased or stayed elevated from 12 to 36 months after TBI, and did not return to pre-injury levels across the 3 year follow-up. These findings highlight the importance of addressing behavioral problems after TBI across an extended time frame.

## Introduction

Exposure to physical trauma during childhood is associated with increases in emotional symptoms and behavior problems in a substantial number of children (1, 2). Among children with physical trauma due to traumatic brain injury (TBI), up to 50% of children are at risk for developing behavior problems and disorders (3). TBI has been linked to both an increase in behavior problems (4–6), and an onset or exacerbation of a variety of psychiatric disorders, including attention deficit/hyperactivity disorder, major depression, post-traumatic stress disorder, and anxiety (7–10). The behavioral and psychiatric problems following TBI are a major source of disability for survivors and a primary cause of family burden. As over 800,000 children seek care for TBI annually, including over 23,000 hospitalizations and 2,500 deaths, disability after TBI is an important public health problem (11).

Post-traumatic emotional symptoms and behavior problems are often assessed dimensionally using rating scales. These scales typically distinguish between internalizing problems such as anxiety and depression that are directed inward, and externalizing problems such as oppositionality or conduct problems directed toward the external environment. In a number of studies, TBI severity is associated with post-traumatic behavioral changes. Ratings completed by parents and/or children indicated greater internalizing and/or externalizing symptoms after moderate to severe TBI than in children with orthopedic injuries (OI) (5, 12) or healthy children (13). Some studies identified greater problems following severe TBI than complicated/mild, moderate, and/or mild TBI for conduct, affective, anxiety, and ADHD problems (5, 6, 12), externalizing problems (14–18), and total behavior problems (19, 20). Conversely, others did not report differences between TBI severity groups in terms of internalizing and/or externalizing problems (21–23). When assessed during adolescence or adulthood, children with a range of TBI severity show elevated risk for internalizing and/or externalizing problems (24–27).

Despite the high incidence and persistence of emotional and behavioral problems following TBI, little is known about the non-injury factors that place children at elevated risk for chronic psychological health concerns. Due to the dearth of methodologically rigorous longitudinal studies, it is unclear whether children's emotional and behavioral profiles stabilize or whether the presence of certain factors contributes to positive outcomes or to a negative cascade and worsening of psychological health over time. Non-injury factors that may place the child at higher risk for poor post-injury functioning include the quality of children's psychological health prior to the injury. Poorer pre-injury adjustment is a risk factor for poorer post-injury adjustment across the spectrum of TBI severity (5, 28). Pre-injury family and environmental factors also significantly influence child behavioral outcomes, such that lower income, lower social and community connectivity, greater family dysfunction, and parental psychiatric symptoms act as vulnerability factors contributing to worse post-injury problems (5, 19, 29–32).

Other child characteristics prior to TBI, such as adequacy of executive functions (EF) supporting cognitive and behavioral self-regulation, may play a key role in shaping outcomes. EF regulate focusing and sustaining attention, resisting distraction, managing frustration, controlling emotional responses, monitoring behavior, considering consequences of behavior, reflecting on past experiences, and planning for the future (33, 34). Although it is well-known that TBI disrupts EF (35–39), there is very limited understanding of how the quality of EF prior to injury shapes behavioral outcomes.

Understanding of multiple factors influencing the long-term trajectory of behavior problems after TBI requires incorporating an assessment of pre-injury behavior and injury comparison groups. Controlling for pre-injury adjustment is essential to discriminate lifetime problems from injury-related changes. It is important to dissociate pre-injury tendencies, such as impulsivity, that may pre-dispose children to injury, from post-injury changes. In addition, using an injured comparison group allows identification of the effects of TBI above and beyond changes that may occur due to the known stresses simply from being injured and receiving medical intervention. The few longitudinal cohort studies controlling for pre-injury behavior and incorporating an orthopedic injury comparison group have identified increases in parent-reported internalizing and externalizing problems across the first year across the spectrum of TBI severity (5, 12, 40).

In this prospective, longitudinal cohort study, we examined parent-reported behavioral outcomes in the largest sample to date of children ages 4–15 with mild to severe TBI relative to an OI comparison group. We used structural equation and growth modeling to examine pre-injury child and family factors shaping long-term internalizing and externalizing problems across the first 3 years after injury. We hypothesized that behavior problems would increase after TBI relative to OI. The level and change over time from pre-injury to 36 months after injury would be related to injury type (TBI or OI), pre-injury child functioning, and family factors. Vulnerability factors including child sex, greater pre-injury EF and adjustment problems, lower income, and family dysfunction were expected to increase the level of emotional symptoms and behavior problems and flatten the trajectory of recovery. Internalizing and externalizing problems were expected to have both shared and unique predictors.

## Materials and Methods

### Participants

**Figure 1:**
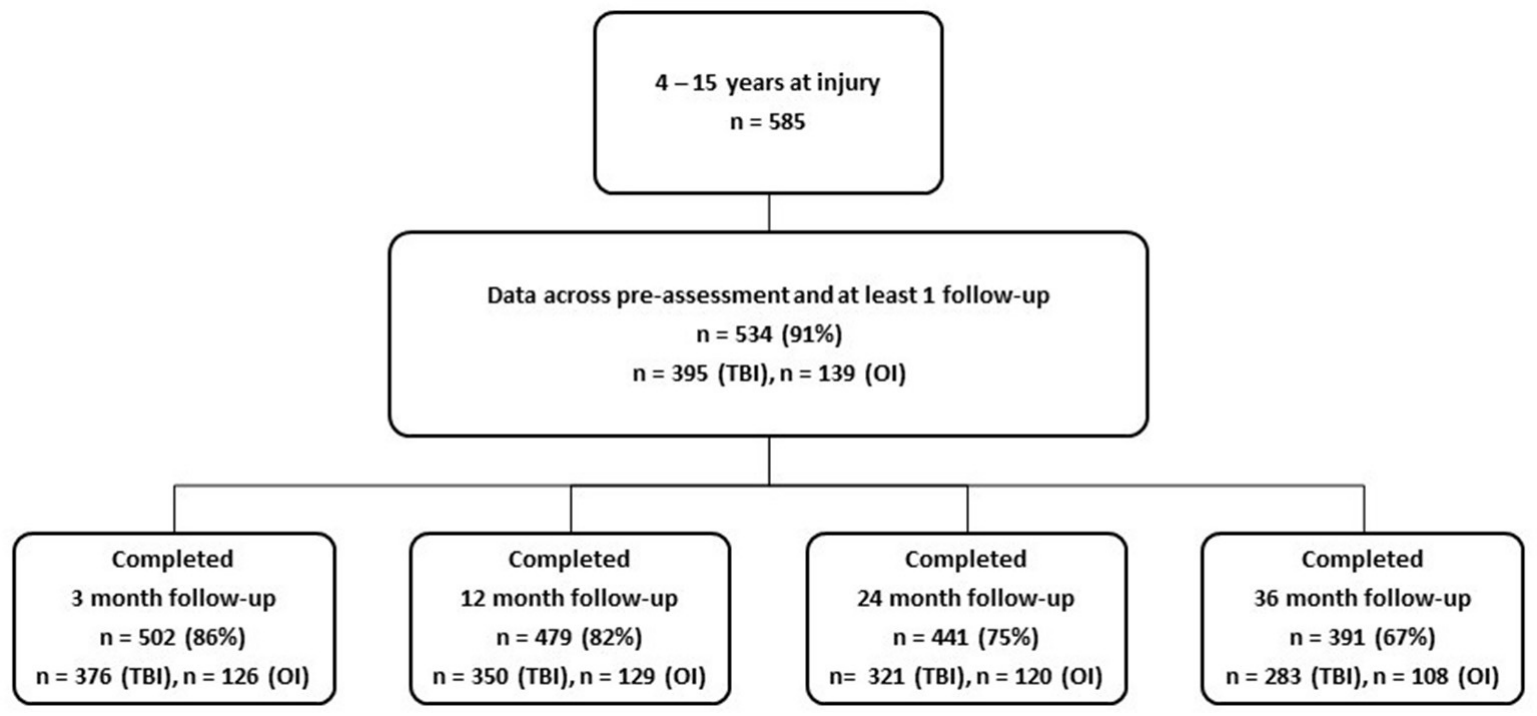
Participation rates across follow-up intervals.

Children ages 0–15 years (*n* = 834) with TBI or OI were recruited for a longitudinal, prospective cohort study from two level 1 pediatric trauma centers, University of Texas Health Science Center at Houston (UTHealth)/Children's Memorial Hermann Hospital and Primary Children's Hospital (PCH) in Salt Lake City, UT. Parents and children provided written permission and assent per IRB guidelines at UTHealth and University of Utah. Children were recruited in the ED or hospital from January, 2013 through September, 2015 sequentially to fill strata of injury type, severity and age group. Exclusionary criteria included the presence of severe developmental delay or psychiatric diagnoses requiring a closed classroom setting. Children 4–15 years old at injury (*n* = 585) were eligible for all measures. Of those, 534 (91%) contributed data across pre-assessment and at least 1 follow-up (see procedures for more details on follow-up data collections). Figure 1 shows the number of TBI and OI participants contributing data at the 3, 12, 24, and 36 month follow-ups. Approximately 67% were retained across all 3 years.

#### Traumatic Brain Injury Group

TBI severity was measured using the lowest Glasgow Coma Scale (GCS) score in the ED assessing motor, eye, and verbal responses (41). TBI was categorized by severity: mTBI was defined as a GCS ≥ 13 in the ED with a GCS of 15 at discharge or after 24 h if hospitalized, and one or more focal signs including a period of transient confusion, loss of consciousness for 30 min or less, and/or transient neurological abnormalities (11). Mild TBI was sub-classified as complicated mild based on CT evidence of an intracranial hemorrhage (42). Moderate and severe TBI were categorized as a GCS of 9–12 and 3–8, respectively. Intubated and sedated children were scored 3T to indicate that the verbal response could not be assessed due to intubation.

#### Orthopedic Injury Group

The OI comparison group included children with an upper or lower extremity long bone fracture without TBI to isolate the effect of TBI from more general injury effects and to account for pre-injury child characteristics, such as impulsivity, that may pre-dispose children to injury. Injury severity was measured with the trauma registrar assigned Abbreviated Injury Scale (AIS) (43).

### Study Design

Parents completed baseline surveys of family demographics, family functioning, social support, and child outcome measures a median of 8 days (IQR: 3, 15) after injury in English or Spanish to represent pre-injury values. Follow-up assessments were collected at 3, 12, 24 and 36 months on-line or by telephone. Clinical and injury variables were abstracted from medical records by study coordinators using standardized forms.

### Measures

All measures are presented in Table 1. Longitudinal trajectories of internalizing and externalizing behavior problems were evaluated using the Strengths and Difficulties Questionnaire (SDQ) Emotional Symptoms and Conduct Problems scales, respectively (44). Parent-reported measures of pre-injury child psychological health and EFs, as well as the family environment, were selected to highlight child behavior and family characteristics potentially related to subacute and chronic behavior problems. All measures are gold standard common data elements recommended for pediatric TBI by the National Institute of Neurologic Disorder and Stroke (51). Child and family variables include psychosocial support factors that are strongly related to positive child outcomes in children facing medical and environmental challenges (52).

**Table 1:** Description of outcome variables and candidate child and family predictors/covariates.

	**Measure description and dependent variables**
**Primary outcomes**	
Emotional Symptoms and Conduct Problems	Scales from Strengths and Difficulties Questionnaire (SDQ) (44), a 25-item behavioral screening questionnaire rated on a Likert scale assessing internalizing and externalizing behavior problems. Satisfactory reliability (α = 0.73) and test-retest stability across 4–6 months (0.62). Higher scores indicate more problems. **Raw scores**.
**Parent ratings of pre-injury child adjustment and health**	
Peer Relationship Problems	SDQ scale assessing difficulty engaging with peers and establishing friendships; Higher scores indicate more problems. **Raw score**.
Prosocial Behavior	SDQ scale evaluating positive behavior and willingness to help others. Higher score indicates fewer difficulties. **Raw score**.
Child Health Questionnaire-PF-28 (CHQ) (45)	Subscales assessing child's health related quality of life based on any limitation in participation. **Role/Social Limitations-Emotional/Behavioral** evaluates impact on school work or activities with friends due to emotional or behavioral factors. **Role/Social Limitations-Physical** assesses limitation in physical activities due to health problems. **Standardized scores** range from 0 to 100; higher scores indicate better functioning.
Post-concussive Symptom Inventory-Parent (46)	Rating of physical, cognitive, emotional, and sleep symptoms often endorsed in healthy samples that are exacerbated by TBI. Twenty items are rated on 7 point Likert scale. Satisfactory internal consistency. (Cronbach's α = 0.78–0.82). Higher scores indicate more symptoms. **Total raw score**.
**Pre-injury child executive functions**	
Behavior Rating Inventory of Executive Functions (BRIEF) (47)	Rating of everyday executive skills involved in behavioral regulation and metacognition. **Inhibit, Emotional Control, Initiate, Working Memory, Plan/Organize, Organization of Materials**, and **Monitor** scales were included. High test-retest reliability (0.82–0.88). Higher scores indicate greater executive dysfunction. **T score**.
ADHD characteristics	The Child Behavior Checklist (CBCL) (48) **Attention-Deficit Hyperactivity Disorder Problems** scale (**T-score**) and the SDQ **Hyperactive/Inattention** scale (**raw score**) assess difficulties regulating attention, excessive activity level, and impulsivity. Higher scores indicate more ADHD symptoms.
**Family environment**	
McMaster Family Assessment Device (FAD) (49)	This scale is composed of 12 items scored from 1 to 4. Items are summed and divided by the total to yield a summary score. Higher scores indicate greater family dysfunction. Cronbach's α = 0.87, test-retest stability across 1 week = 0.66–0.76. **Total score**.
Social Capital Index (50)	Sum of factors promoting positive adaptation, including marital support, personal social support, family size, neighborhood support, spiritual community. Scores range 1–5 with higher scores representing more support. **Total score**.
Income and education	Families self-reported their educational attainment and income category; we calculated income relative to poverty level by family size based on federal norms.

### Data Reduction

In order to create a latent EF factor, confirmatory factor analysis was used to combine EF data including BRIEF variables, SDQ Hyperactivity and CBCL ADHD Problems at the pre-injury time point as is commonly done (53, 54). We examined fit *via* the Comparative Fit Index (CFI), and Root Mean Square Error of Approximation (RMSEA). CFIs >0.90, and RMSEAs <0.08 were used to evaluate whether a model demonstrated “acceptable fit” (55). Initially the model did not fit well (CFI = 0.80, RMSEA = 17). However, modification indices indicated some measurement variance associated with the BRIEF. We allowed residual correlations among subscales for the BRIEF which improved fit to acceptable levels (RMSEA = 0.07, CFI = 0.99). Given field results suggesting that a two factor (hot/affective regulation and cool/decontextualized) may fit better, we also fit a 2 factor model with residual correlations for the BRIEF. We defined hot regulation *via* the BRIEF emotional control and inhibit scales as well as the ADHD subscale of the CBCL. All other scales defined the cool/de-contextual factor. The two factor did not fit as well (RMSEA = 0.13, CFI = 0.92); thus, we retained the one factor.

### Statistical Analyses

First, we wanted to identify any significant differences between the OI and the different TBI groups. To do this, we performed within time point ANOVAs with planned group comparisons at all time points. The TBI and OI groups were similar at the pre-injury time point, but there were consistent, significant differences between the TBI group and OI group at most subsequent time points. When examining differences among the TBI severity groups, we first combined complicated mild and moderate TBI into one group due to the small number of children with moderate TBI (42). However, the ANOVAs with planned comparisons indicated that for both Emotional Symptoms and Conduct Problems, there were no significant differences between the TBI groups across all time points. Therefore, TBI was collapsed into a single group for the remaining analyses.

Next, we evaluated how best to model change in SDQ Emotional Symptoms and Conduct Problems between pre-injury and 36-months post. Five growth models were fit separately to the Emotional Symptoms and Conduct Problem data: linear, quadratic, and cubic growth as well as two spline models: a linear-linear spline model and a quadratic-linear spline model with the knot point set at 12-month post. This approach allowed us to (i) accurately model expected slope changes related to disruption and recovery in child functioning around an injury, and (ii) build a larger model focused on child and family level predictors that affect the injury related change process. Fit statistics including the Akaike Information Criterion (AIC), the adjusted Bayesian Information Criterion (aBIC), RMSEA, and CFI, were used to evaluate model fit (55). For the cubic and quad-linear spline models, the variance for the cubic term, and the quadratic term, respectively, were set to zero to ensure enough degrees of freedom to estimate the model. Note even with variance (random effect) fixed, the mean for each of these terms (fixed effect) is still estimated and mean change across predictors can also be estimated. Spline models were used because they are capable of modeling different phases of change by including more than one slope factor. For both spline models, the knot point was set to 12-months, thus the spline models included separate parameters for the slope to capture the change from pre-injury to 12 months post-injury and then from 12 months post to 36-month post-injury. This is a theoretically significant point as substantial recovery occurs by 12 months after TBI (35) and our goal was to evaluate factors influencing the recovery trajectory after 12 months. Models were performed in Mplus 8.2 using maximum likelihood estimation (56) with an ML estimator for all analyses to account for missing data.

#### Injury Predictor

After building the growth curve models, we then included injury type as a predictor of the level of both outcomes at 12-months post-injury and of the change parameters. Our first hypothesis is that internalizing and externalizing problems would increase more after TBI relative to OI. Including injury first allowed us to evaluate and describe the main effect of injury on both the increase/decrease in change and the rate of change, as well as differences in levels of the behavior.

#### Child and Family Predictors

After the inclusion of injury type, we added demographic, pre-injury child characteristics (latent executive factor, parent ratings of adjustment and health) and family environment predictors to the model. These were added simultaneously as we did not have strong hypotheses regarding order of effects. Simultaneous inclusion allowed us to look at each predictor while controlling for the influence of all other predictors.

## Results

### Participants

The cohort consists of 395 children with TBI: 146 mild, 132 complicated mild, 28 moderate, and 89 with severe TBI. There are 139 children with OI. As noted above, there were no demographic or pre-injury differences between the TBI severity groups. Additionally, there were no differences among the TBI severity groups on the primary SDQ outcomes at any time point. Consequently, they were combined into an overall TBI group. Comparison of baseline demographic variables, child characteristics, and family environment indicated no significant differences across TBI and OI groups (Table 2).

**Table 2:** Comparison of baseline demographic variables, child characteristics, and family environment by injury type.

	**Injury type**		**Injury type comparison**
	**Traumatic brain (*n* = 395)**	**Orthopedic (*n* = 139)**	*p*-value^*^
**DEMOGRAPHIC** ***n*** **(%)**			
**Enrollment site**: Utah	229 (58%)	76 (55%)	0.50
**Age** (years): mean (SD)	9.04 (4.24)	8.57 (4.11)	0.26
**Child sex**: Female	142 (36%)	50 (36%)	0.99
**Race/ethnicity**			
•Hispanic or Latino	100 (26%)	41 (30%)	0.75
•White	233 (60%)	76 (55%)	
•Black	30 (8%)	10 (7%)	
•Other/mixed race	27 (7%)	11 (8%)	
**Preferred language: Spanish**	42 (11%)	20 (14%)	0.23
**Parent education**			
•Less than HS	38 (10%)	16 (11%)	
•HS diploma or GED	85 (22%)	19 (14%)	
•Vocational training/some college	144 (37%)	37 (28%)	0.10
•Bachelor's degree	74 (19%)	47 (34%)	
•Advanced degree	53 (13%)	20 (14%)	
**Parents married**	281 (75%)	95 (25%)	0.60
**Either parent employed**	363 (92%)	123 (88%)	0.23
**Income at or below the poverty line**	97 (27%)	31 (23%)	0.65
**CHILD CHARACTERISTICS** ***M*** **(SD)**			
**Adjustment and symptoms**			
•SDQ peer problems	1.39 (1.55)	1.30 (1.49)	0.59
•SDQ prosocial	8.52 (1.82)	8.18 (2.96)	0.10
•CHQ physical restraints	98.33 (8.99)	97.97 (13.95)	0.75
•CHQ emotion/beh. restraints	95.10 (15.40)	95.11 (16.60)	0.99
•PCSI total	4.72 (9.86)	4.29 (9.06)	0.68
**Executive function**			
•BRIEF inhibit	49.19 (11.46)	48.82 (9.97)	0.74
•SDQ hyper	2.98 (2.60)	2.63 (2.22)	0.20
•CBCL ADHD	54.23 (6.30)	53.81 (5.92)	0.49
•BRIEF initiate	47.67 (10.50)	47.90 (10.16)	0.85
•BRIEF monitor	45.59 (11.15)	46.21 (10.73)	0.63
•BRIEF materials	48.63 (9.99)	47.55 (10.32)	0.35
•BRIEF planning	47.41 (11.06)	47.32 (10.30)	0.94
•BRIEF memory	48.99 (11.28)	48.63 (10.65)	0.74
**FAMILY ENVIRONMENT** ***M*** **(SD)**			
**Family Assessment Device**	1.52 (0.45)	1.49 (0.47)	0.50
**Social Capital Index**	3.47 (1.04)	3.60 (1.00)	0.23

* *p-value is associated with either the F-value in an ANOVA looking at injury type (TBI overall vs. OI) comparing continuous variables, or a chi-square value in tests comparing dichotomous or categorical variables*.

*SDQ, Strengths and Difficulties Questionnaire; CHQ, Child Health Questionnaire; PCSI, Post-Concussion Symptom Inventory; BRIEF, Behavior Rating Inventory of Executive Functions*.

### Raw and Fitted Change in Outcome Scores

**Figure 2:**
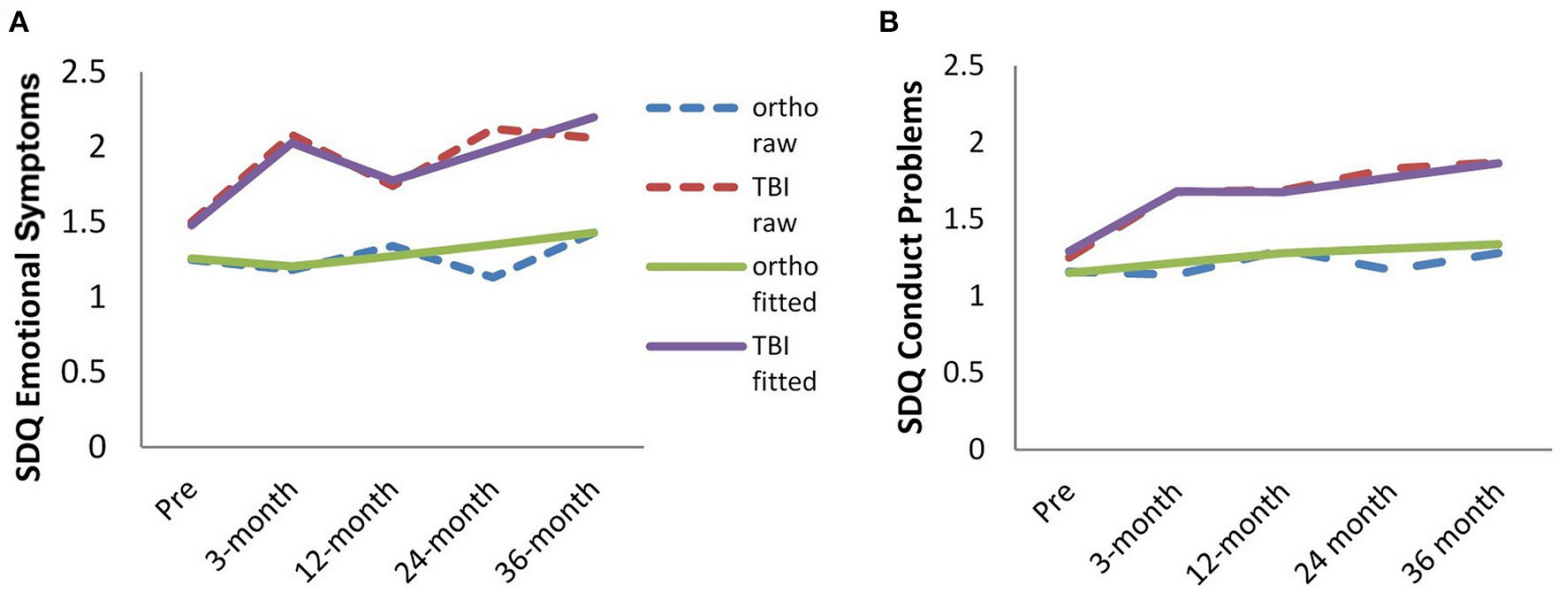
Raw and fitted change in Emotional Symptoms **(A)** and Conduct Problems **(B)** scores from pre-injury through 36-months post-injury for TBI and OI groups.

Descriptive statistics and ANOVA group comparisons of SDQ Emotional Symptoms and Conduct Problem outcomes for TBI and OI groups at each time point are in Table 3. Correlations between these variables are presented in Supplementary Table 1. Ratings across all time points met assumptions of normality (57, 58), ensuring parametric analyses such as ANOVA and subsequent SEM modeling were appropriate. Pre-injury ratings did not differ significantly by group. The TBI group had higher scores than the OI group on both measures at the 3, 24, and 36 month follow-ups. Groups did not differ at the 12 month interval. Figures 2A,B shows the longitudinal trajectory of the raw and fitted scores by group.

**Table 3:** Descriptive statistics and comparisons of Strengths and Difficulties subtest scores by injury type and time point.

	**SDQ Emotional Symptoms**					**SDQ Conduct Problems**				
	**Orthopedic injury**		**TBI**			**Orthopedic injury**		**TBI**		
**Time point**	***n***	***M* (SD)**	***n***	***M* (SD)**	***F* (*p*)**	***n***	***M* (SD)**	***n***	***M* (SD)**	***F* (*p*)**
Pre-injury	116	1.25 (1.43)	319	1.50 (1.91)	1.05 (0.37)	116	1.16 (1.52)	318	1.25 (1.66)	1.53 (0.21)
3-month post	108	1.18 (1.44)	315	2.08 (2.22)	5.82 (<0.001)	108	1.14 (1.17)	315	1.68 (1.95)	3.12 (<0.05)
12-month post	119	1.34 (1.82)	336	1.74 (2.05)	1.19 (0.31)	119	1.30 (1.77)	336	1.69 (2.00)	1.47 (0.22)
24-month post	119	1.13 (1.59)	319	2.12 (2.46)	5.61 (<0.001)	120	1.17 (1.61)	319	1.83 (2.01)	3.72 (<0.05)
36-month post	107	1.43 (1.89)	277	2.06 (2.24)	2.29 (0.08)	107	1.28 (1.64)	279	1.87 (2.09)	3.23 (<0.05)

### Growth Curve Models

Fitted data from the 5 growth models evaluated were examined to inform the best model representing the raw data at each time point. Latent growth fit statistics for each model are in Supplementary Table 2. The quadratic-linear spline model best fit both outcome variables, indicating quadratic change between pre-injury and 12-month post, and linear change between 12- and 36-month post, with the level estimates at the knot point (12-months post). The addition of the quadratic term (from pre-injury to 12-months pots) for Emotional Symptoms describes a period of rapid increase followed by rapid decrease. For Conduct Problems, the quadratic captures the rate change with a rapid increase that levels off. The linear term fit well for both outcomes from 12- to 36-months.

#### Emotional Symptoms

##### Injury Type

**Figure 3:**
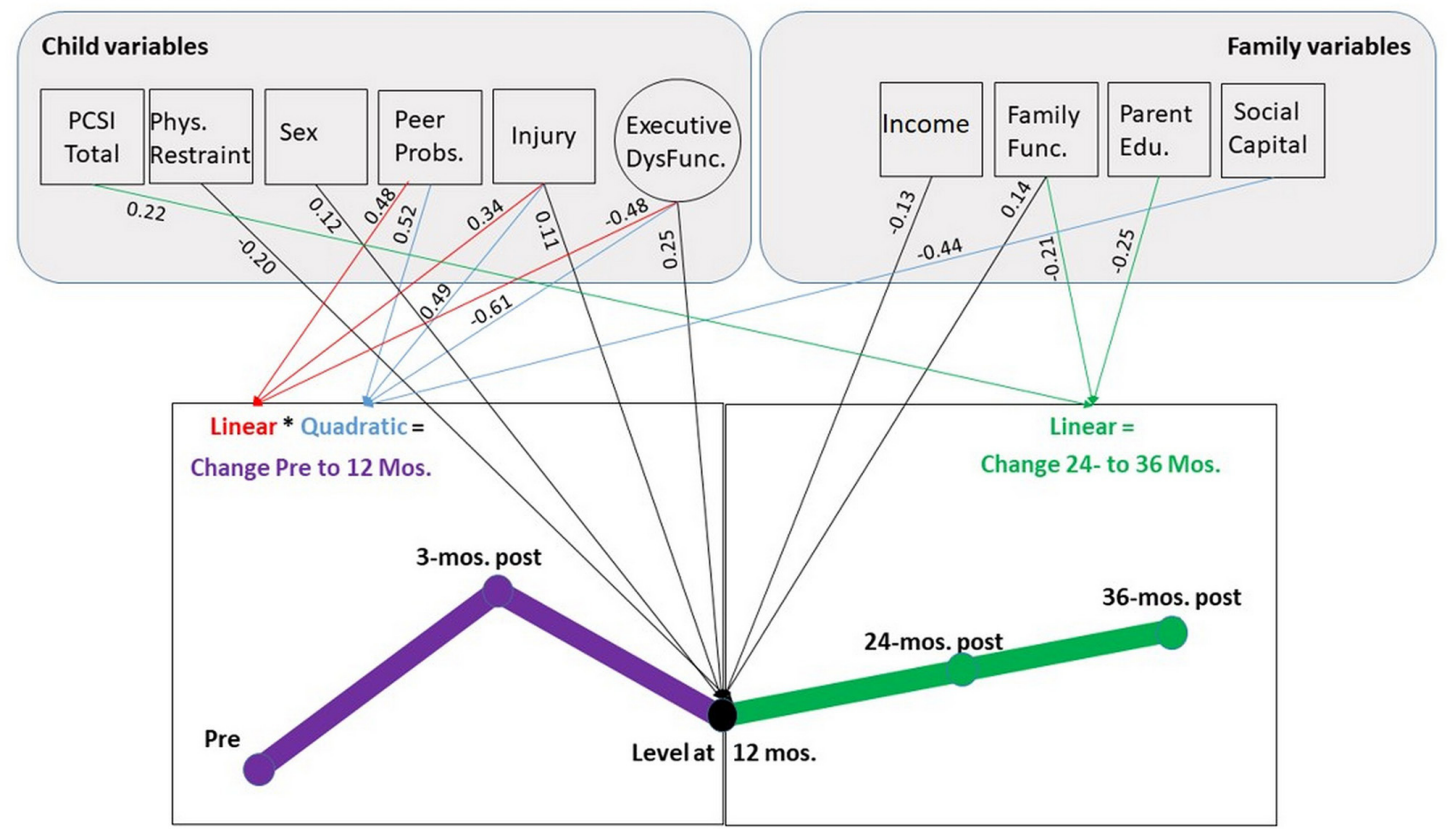
SEM model of change in Emotional Symptoms as predicted by injury type and pre-injury EFs, child and family factors. Predictor variables with no significant relationships, correlations between pre-injury variables, variances, and residual variances are suppressed. Direct relationships are denoted by arrows. Circles represent latent constructs. Boxes represent measured constructs. Coefficients are shown for measures at 12 months, linear and quadratic change. PCSI, peer problems, executive dysfunction, and family function scores indicate more problems and are expected to positively associated with emotion symptoms. Higher physical restraint and social capital scores indicate better functioning/ more social connections and are expected to negatively associate with Emotional Symptoms. Sex is coded 0 for males and 1 for females. Injury is coded 0 for the orthopedic group and 1 for the TBI group. Income is coded such that higher values indicate less poverty. Parent education is coded such that higher values indicate greater educational attainment.

The level and change parameters for injury type and all pre-injury predictors of Emotional Symptoms are presented in Table 4 and Figure 3. Injury type was a significant predictor of the level at 12 months post-injury (*p* < 0.05) such that children with TBI reported 0.11 of a standard deviation increase in Emotional Symptoms (approximately a half a point increase on the SDQ compared to orthopedic peers). In contrast, children with an OI demonstrated few changes in Emotional Symptoms at the 12 month time point. Injury type was important for both the pre-injury to 12-months linear and quadratic change parameters (linear β = 0.34, *p* < 0.05, quadratic β = 0.49, *p* < 0.01) indicating that Emotional Symptoms in children with TBI demonstrated a rapid increase after injury and then declined prior to the 12-month time point, whereas OI group demonstrated little to no changes in Emotional Symptoms.

**Table 4:** Summary of injury type and pre-injury child and family predictors of change in Emotional Symptoms.

**Predictor**	**SDQ Emotional Symptoms**							
	**Level @ 12 months**		**Linear change: pre-injury–12 months**		**Quadratic change: pre-injury−12 months**		**Linear change: 12 month−36 months**	
	**Coef (se)**	***P***	**Coef (se)**	***P***	**Coef (se)**	***P***	**Coef (se)**	***p***
**Injury type**								
TBI	0.11 (0.05)	0.02^*^	0.34 (0.15)	0.02^*^	0.49 (0.17)	<0.01^*^	0.09 (0.07)	0.21
Orthopedic	(Ref)	(Ref)	(Ref)	(Ref)	(Ref)	(Ref)	(Ref)	(Ref)
**Child characteristics**								
•Age	−0.04 (0.05)	0.46	0.12 (0.18)	0.50	0.11 (0.21)	0.59	−0.02 (0.08)	0.76
•Sex (male = 0)	0.12 (0.05)	0.02^*^	−0.09 (0.16)	0.56	−0.08 (0.19)	0.69	−0.01 (0.08)	0.87
•SDQ peer problems	0.09 (0.06)	0.14	0.48 (0.16)	<0.01^*^	0.52 (0.19)	0.01^*^	0.05 (0.09)	0.59
•SDQ prosocial	0.10 (0.08)	0.07	0.05 (0.18)	0.77	0.14 (0.21)	0.51	−0.08 (0.09)	0.39
•CHQ physical restraints	−0.20 (0.05)	<0.001^*^	0.23 (0.16)	0.15	0.15 (0.19)	0.43	−0.003 (0.09)	0.98
•CHQ emotion/beh restraints	−0.04 (0.06)	0.47	−0.14 (0.17)	0.42	−0.18 (0.20)	0.36	0.13 (0.08)	0.13
•PCSI total	0.09 (0.06)	0.15	−0.02 (0.19)	0.92	−0.28 (0.22)	0.21	0.22 (0.11)	0.04^*^
**EF factor**•BRIEF inhibit λ = 0.80•SDQ hyperactivity λ = 0.86•CBCL ADHD problems λ = 0.84•BRIEF initiate β = 0.69•BRIEF monitor β = 0.67•BRIEF organize materials β = 0.54•BRIEF planning β = 0.74•BRIEF memory β = 0.82	0.25 (0.07)	<0.001^*^	−0.48 (0.19)	<0.01^*^	−0.61 (0.22)	<0.01^*^	−0.09 (0.11)	0.41
**Family environment**								
•Parent highest ed.	0.10 (0.06)	0.13	0.22 (0.21)	0.30	0.34 (0.25)	0.15	−0.25 (0.10)	0.02^*^
•Family Assessment Device	0.14 (0.05)	0.01^*^	−0.16 (0.17)	0.35	−0.18 (0.20)	0.38	−0.21 (0.08)	0.02^*^
•Social Capital Index	−0.08 (0.05)	0.12	−0.31 (0.17)	0.06	−0.44 (0.19)	0.02^*^	−0.13 (0.08)	0.11
•Income	−0.13 (0.06)	0.02^*^	0.13 (0.19)	0.50	−0.09 (0.22)	0.68	0.18 (0.09)	0.05^†^
•Language (1 = Spanish)	−0.10 (0.06)	0.07	0.24 (0.18)	0.18	0.17 (0.22)	0.43	−0.01 (0.08)	0.82

*CBCL, Child Behavior Checklist; CHQ, Child Health Questionnaire; EF, Executive Function; PCSI, Post-Concussion Symptom Inventory; SDQ, Strengths and Difficulties Questionnaire*.

† *p = 0.05*,

* *p < 0.05*.

The third change parameter examined linear change from 12- to 36-months post-injury. The overall change with no predictors in the model was significant β = 0.26, *p* < 0.05. This means that for every 1 year, there is a 0.26 standard deviation increase in Emotional Symptoms. However, injury type was not a significant predictor of this change (β = 0.09, *p* = 0.21). Both injury groups had linear increases, even though the change in scores for the OI group was minor. Across the extended follow-up, the TBI group did not return to pre-injury levels of Emotional Symptoms and remained elevated above OI peers.

##### Pre-Injury Child and Family Predictors of Change

Briefly, greater Emotional Symptoms 12-month after injury were predicted by TBI, female sex, as well as greater pre-injury executive dysfunction and physical limitations measured on the CHQ. Family burden also played a key role with higher levels of family dysfunction and lower income associated with higher levels of emotional difficulties. The linear and quadratic slope from pre-injury status to 12 months post-injury were predicted by TBI, less pre-injury executive dysfunction, and higher peer problems in conjunction with lower family social capital. The direction of the EF parameter was unexpected; higher levels of pre-injury executive dysfunction dampened the increase and rate of change in Emotional Symptoms. Higher social capital was negatively associated with the quadratic parameter, suggesting that more social support dampened the rapid rise and fall of Emotional Symptoms in the first 12-months post.

The linear slope from 12 to 36 months was not predicted by injury type or most child characteristics. The one exception was that higher total PCSI symptoms prior to injury was associated with an increase in Emotional Symptoms. For pre-injury family factors, higher levels of parent education and family dysfunction dampened the increase in Emotional Symptoms from 12 to 36 months.

#### Conduct Problems

##### Injury Type

Whether a child sustained a TBI or OI was also a significant predictor of the level of Conduct Problems at 12 months post-injury (β = 0.11, *p* < 0.05). Children with TBI reported 0.11 of a standard deviation increase in Conduct Problems (a little under a half a point increase compared to OI) at the 12 month time point. Type of injury was not related to the linear change parameter from pre-injury to 12-months, but it was related to the quadratic parameter (linear β = 0.23, *p* = 0.16, quadratic β = 0.36, *p* < 0.05). As can be seen in Figure 2B, this suggests a significant difference in the rate of change as Conduct Problems increase particularly between pre-injury and 3-months after TBI.

Across the long-term follow-up from 12 to 36 months, the base model with no predictors found an increase in the slope that approached significance, β = 0.21, *p* = 0.09. Injury type was not a significant predictor of change (β = 0.11, *p* = 0.48), with problems remaining elevated after TBI compared to the OI group across the follow-up. While a linear model fit best, it did not suggest upward linear change. Rather, a flatline linear constant model may best characterize the slope across both groups.

##### Pre-Injury Child Characteristics and Family Predictors of Change

**Figure 4:**
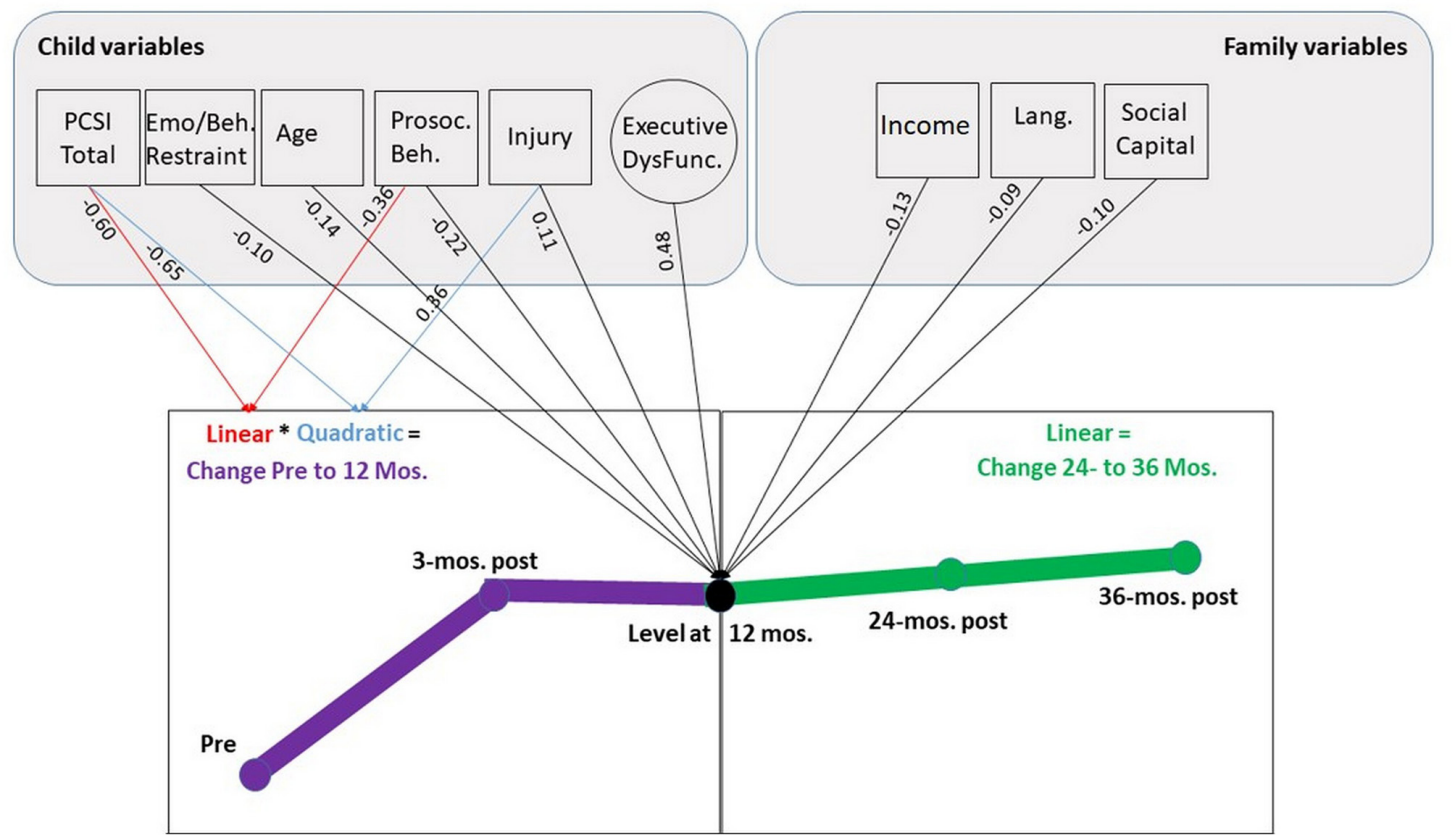
SEM model of change in Conduct Problems as predicted by child and family factors prior to injury. Predictor variables with no significant relationships, correlations between pre-injury variables, variances, and residual variances are suppressed. Higher PCSI and executive dysfunction scores indicate more problems and are expected to positively associated with conduct problems. Higher emotion and behavior restraints, pro-social behavior, and social capital scores indicate better functioning/more social connections and are expected to negatively associate with Conduct Problems. Injury is coded 0 for the orthopedic group and 1 for the TBI group. Income is coded such that higher values indicate less poverty. Language is coded 0 = prefer English, and 1 = prefer Spanish. Parent education is coded such that higher values indicate greater educational attainment.

The level and change parameters on all pre-injury predictors are presented in Table 5 and Figure 4. At 12-months post-injury, seven child and family variables predicted the level of Conduct Problems. Higher scores were predicted by TBI (β = 0.11, *p* < 0.01) and executive dysfunction (β = 0.48, *p* < 0.001). For every one factor unit increase in executive dysfunction, there was a 0.48 (or nearly half a standard deviation) increase in Conduct Problems. Other child predictors were younger age (β = −0.14, *p* < 0.01), more CHQ emotional/behavioral restraints, and lower SDQ prosocial behaviors. Higher scores on the CHQ emotional/behavioral restraints scale (with higher indicating better functioning) were associated with a 0.10 standard deviation reduction in 12-months post-injury Conduct Problems. Likewise lower levels of pro-social behavior were associated with a 0.22 standard deviation increase in Conduct Problems at 12-months post-injury. Among family characteristics, higher Conduct Problems were predicted by lower social capital (β = −0.10, *p* < 0.05), being an English language speaker (β = −0.09, *p* < 0.05), and lower income (β = −0.13, *p* < 0.05).

**Table 5:** Summary of pre-injury child and family predictors of change in Conduct Problems.

**Predictor**	**SDQ Conduct Problems**							
	**Level @ 12 months**		**Linear change: pre-injury−12 months**		**Quadratic change: pre-injury−12 months**		**Linear change: 12 month−36 months**	
	**Coef (se)**	***p***	**Coef (se)**	***P***	**Coef (se)**	***p***	**Coef (se)**	***p***
**Injury type**								
TBI	0.11 (0.04)	0.01^*^	0.23 (0.17)	0.16	0.36 (0.17)	0.03^*^	0.11 (0.15)	0.48
Orthopedic	(Ref)	(Ref)	(Ref)	(Ref)	(Ref)	(Ref)	(Ref)	(Ref)
**Child characteristics**								
•Age	−0.14 (0.05)	<0.01^*^	−0.14 (0.19)	0.47	−0.20 (0.20)	0.31	−0.06 (0.15)	0.71
•Sex (male = 0)	0.05 (0.04)	0.28	−0.16 (0.17)	0.34	−0.12 (0.19)	0.50	−0.22 (0.17)	0.20
•SDQ peer problems	0.07 (0.05)	0.20	0.02 (0.20)	0.93	0.01 (0.21)	0.96	−0.23 (0.22)	0.28
•SDQ pro-social	−0.22 (0.05)	<0.001^*^	−0.36 (0.18)	<0.05^*^	−0.37 (0.19)	0.06	−0.02 (0.18)	0.90
•CHQ physical restraints	−0.07 (0.05)	0.13	0.13 (0.18)	0.49	0.08 (0.19)	0.68	−0.11 (0.20)	0.59
•CHQ emotion/beh. restraints	−0.10 (0.05)	0.04^*^	−0.08 (0.19)	0.68	−0.11 (0.20)	0.56	0.11 (0.17)	0.52
•PCSI total	0.09 (0.06)	0.11	0.60 (0.19)	0.001^*^	−0.65 (0.19)	<0.01^*^	−0.46 (0.29)	0.11
**EF factor** •Brief inhibit λ = 0.81 •SDQ hyperactivity λ = 0.85 •CBCL ADHD problems λ = 0.85 •Brief initiate β = 0.69 •Brief monitor β = 0.67 •Brief organize materials β = 0.55 •Brief planning β = 0.74 •Brief working memory β = 0.81	0.48 (0.06)	<0.001^*^	−0.31 (0.22)	0.16	0.39 (0.23)	0.09	−0.20 (0.23)	0.39
**Family environment**								
•Parent highest ed.	−0.03 (0.06)	0.63	0.14 (0.20)	0.48	0.20 (0.24)	0.41	0.14 (0.20)	0.49
•Family Assessment Device	−0.01 (0.05)	0.82	0.08 (0.19)	0.68	0.06 (0.20)	0.78	−0.16 (0.17)	0.37
•Social Capital Index	−0.10 (0.05)	0.04^*^	−0.07 (0.19)	0.07	−0.19 (0.19)	0.32	−0.09 (0.16)	0.59
•Income	−0.13 (0.06)	0.02^*^	0.02 (0.18)	0.92	−0.32 (0.21)	0.14	0.02 (0.18)	0.92
•Language (1 = Spanish)	−0.09 (0.05)	<0.05^*^	0.07 (0.20)	0.74	−0.09 (0.21)	0.69	−0.06 (0.17)	0.71

*CBCL, Child Behavior Checklist; CHQ, Child Health Questionnaire; EF, Executive Function; PCSI, Post-Concussion Symptom Inventory; SDQ, Strengths and Difficulties Questionnaire*.

* *p < 0.05*.

The only predictors of the rate of change in Conduct Problems from pre-injury to 12 months were children's pre-injury prosocial behaviors (linear β = −0.36, *p* < 0.05, quadratic: β = −0.37, *p* = 0.06) such that higher levels of pro-social behavior predicted less of an increase (and a dampened rate of increase). Similarly, higher pre-injury concussion-type symptoms were actually associated with a decrease in the change and rate of change of Conduct Problems. Pre-injury family characteristics predicted neither linear nor quadratic change across the first year after injury.

The linear slope from 12 to 36 months was not predicted by injury type, child characteristics, or family characteristics.

## Discussion

In this longitudinal prospective cohort study, we followed the recovery of Emotional Symptoms and Conduct Problems as proxies of internalizing and externalizing behavior problems across the first 3 years after pediatric TBI vs. OI. We found that among children with TBI, both Emotional Symptoms and Conduct Problems were significantly elevated at 12 months and did not recover to pre-injury levels over the subsequent 2 years relative to the OI group. This is in contrast to children with OI, who showed minimal increases in either Emotional Symptoms or Conduct Problems across time. Our findings suggest that the elevations in problems may be attributed to the TBI and not to the experience of sustaining an injury *per se*. Growth models revealed that TBI was associated with a steeper non-linear increase in Emotional Symptoms and Conduct Problems from pre-injury to 12 months and increased level at 12 months relative to the OI group. Growth from 12 to 36 months was relatively flat in both groups, which suggests that children do not have increasing problems, but also do not recover.

Consistent with our hypotheses, we identified both shared and unique vulnerability factors affecting the level and change in Emotional Symptoms and Conduct Problems over time. Shared vulnerability factors included experiencing a TBI and having more pre-injury executive dysfunction. The increase in Emotional Symptoms and Conduct Problems at 12 months is consistent with prior longitudinal studies reporting increased internalizing and/or externalizing problems during the year after TBI after accounting for pre-injury status (12, 19, 35, 59). There is limited information regarding whether internalizing and externalizing problems increase systematically as TBI severity increases. In our sample, there were no differences in behavior problems between patients with mild, moderate, or severe TBI at any timepoints Although few studies have examined behavior problems across the spectrum of TBI severity, there is evidence that children with mild TBI (19, 59) and complicated-mild to severe TBI (5, 12) are at increased risk.

The centrality of EF as a predictor of both internalizing and externalizing outcomes is a novel finding. Although EF are key markers predicting future attainments across a variety of ages and developmental conditions (60, 61), the influence of pre-injury EF on outcomes has rarely been considered. Children who experience difficulties in inhibition, working memory, and attention-deficit hyperactivity problems appear to be at elevated risk for a broad range of behavior problems after TBI. After TBI in early childhood, Narad et al. found that greater pre-injury executive dysfunction was associated with greater likelihood of clinically significant EF symptoms persisting up to 7 years after injury (39). While It is unclear to what extent pre-existing EFs problems shape post-injury cognition and behavior in school-aged children and adolescents, there is a growing body of evidence showing significant and persistent adverse effects of TBI on EFs across the first 2 years after injury, with greater disruption following severe TBI than less severe injuries (35, 38). Long term follow up studies examining EF outcomes from 3 to 10 years after injury have also shown persistent problems, with EFs not returning to pre-injury levels (35, 62–64). Executive dysfunction after TBI has serious consequences for post-traumatic adjustment (65) and contributes to poorer educational outcomes (66, 67) and reduced social competence (68). TBI may exacerbate pre-existing EF problems in children and may magnify vulnerability to an array of poorer outcomes. Future studies should disentangle the effects of pre-existing vs. acquired EF on both cognitive and behavioral outcomes.

In addition to TBI and EFs, specific pre-injury child and family factors uniquely predicted the rate of change and level of Emotional Symptoms during the first year after TBI. After controlling for pre-injury ratings, girls had increased Emotional Symptoms 12 months post-injury. There were no differences in the rate of change over time for girls and boys. This finding is consistent with prior literature suggesting vulnerability of girls after both TBI (10, 69–72) and the broader category of pediatric acquired brain injury (73). Similarly, increased internalizing and ADHD symptoms were noted in girls compared to boys when assessed during the first year after injury after accounting for pre-injury ratings (5). Longer term follow-up studies found increases in internalizing symptoms over several years after TBI in both sexes; additionally, younger boys showed greater oppositional defiant problems and older girls showed greater ADHD symptoms (6). Developmentally, internalizing behavior problems tend to increase more in girls than boys during adolescence (74, 75), while externalizing problems are elevated in boys relative to girls (76). However, we did not identify vulnerability of boys to increases in either outcome domain. Scott et al. assessed adulthood outcomes after pediatric TBI. They found that women reported more internalizing problems and men reported more externalizing problems (26). To better understand the influence of sex, age, and time since injury, future studies should examine the risk of both internalizing and externalizing problems in boys and girls across different developmental stages.

In our sample, increased Emotional Symptoms were also predicted by specific pre-injury child factors including problems in peer relations and physical limitations such as low energy that reduced participation in everyday activities. Interestingly, while poor EF was associated with elevated Emotional Symptoms at 12 months, it was also associated with a slower rate of increase in symptoms from injury to 12 months. It is possible that children with greater pre-injury executive dysregulation are less likely to develop the types of somatic, anxiety, and depression symptoms tapped by this subtest. Such children may be more likely to develop externalizing than internalizing behaviors over time. This is indicated by the positive quadratic change parameter for the EF factor that approached significance in the Conduct Problem analysis. Family risk factors, including increased family dysfunction, as well as lower parental education, income, and social integration into the community were also important predictors of increased Emotional Symptoms. The influence of family factors on a variety of outcomes after TBI is supported by prior literature (29, 77, 78). Changes across long-term follow-ups were largely related to both pre-existing post-concussion-like symptoms and family factors rather than injury factors. Additional work should characterize whether prior emotional, somatic, or fatigue concussion-like symptoms drive this relation. Given the contribution of family factors and the growing recognition of the contribution of family and parenting factors to child behavioral outcomes after TBI (6, 79), psychological interventions including a family component are appropriate targets for intervention.

Conduct Problems also had unique vulnerability factors. A higher level of Conduct Problems at year 1 was found in younger children and children with more prior emotional/behavioral difficulties and lower prosocial behaviors. As expected, family indicators were also related to the level of Conduct Problems 1 year post-injury; higher social capital, higher income and Spanish as preferred language were associated with fewer Conduct Problems. In contrast to Emotional Symptoms, change from pre-injury to 1 year after injury was related to unique child characteristics. Poorer prosocial behavior was associated with increased Conduct Problems while more PCS-like symptoms were associated with less increase or a dampening of Conduct Problems. Also differing from Emotional Symptoms, no variables predicted change in Conduct Problems over the extended follow-up. This suggests that Conduct Problems became stable deficits by 1 year after TBI and showed no trend toward recovery. Our findings are similar to Ryan et al., who found a high rate of externalizing behavior problems persisting into young adulthood that were not related to either TBI severity or to family characteristics (80). Previous studies have linked EFs to changes in externalizing problems over time. Additionally, parent characteristics and parenting practices are associated with child externalizing problems after TBI (31, 81). Consequently, comprehensive intervention strategies that target parenting practices, in addition to addressing EFs and externalizing symptoms, may be fruitful targets to improve outcomes after TBI. Evidence-based programs that can be delivered online and customized to family structure offer advantages for improving family outcomes (82).

## Conclusions and Limitations

Our findings should be viewed in relation to study limitations. We included only parent-reported outcomes, which may result in a limited view of behavior concerns, especially internalizing problems. Our findings are based on behavior ratings and do not incorporate any direct measures of child abilities or characteristics. Although we used well-validated measures that are common data elements for TBI outcomes, all measures were collected online or by telephone, which may differ from in-person evaluations. Our intent was to examine pre-injury predictors of outcome. However, including post-injury child and family changes may provide additional insight into factors influencing the trajectory of long-term outcomes.

Strengths of the study include the longitudinal, prospective design including a large and well-characterized sample with a broad spectrum of TBI severity and age range. Statistical approaches incorporated multivariable predictors in a structural equation modeling framework and identified factors contributing to the level and change in internalizing and externalizing behavior problems across the first 3 years after TBI. We emphasized a range of pre-injury child and family characteristics as potential influences on long-term adjustment. Including pre-injury EF as a predictor of outcomes is novel and has potential to improve understanding of child vulnerability and protective factors.

In conclusion, our findings indicated significant increases in both internalizing and externalizing behavior problems after TBI. This increase was stable across the prospective extended 3 year follow-up, indicating that problems accelerated across the first year after TBI and then either stayed stable or increased from 12 to 36 months after TBI, resulting in chronic behavior changes. We identified both shared and unique influences shaping behavior. During the first year after injury, shared vulnerability factors for internalizing and externalizing behavior problems included TBI, less favorable pre-injury child EFs, and poverty. All other child and family factors were uniquely related to the level of Emotional Symptoms and Conduct Problems. Our finding that long term internalizing problems were more strongly related to family factors and that externalizing problems were more strongly related to child factors suggests that personalized approaches to child and family intervention may be warranted.

## Data Availability Statement

The raw data supporting the conclusions of this article will be made available by the authors, without undue reservation.

## Ethics Statement

The studies involving human participants were reviewed and approved by Committee for the Protection of Human Subjects at University of Texas Health Science Center and at University of Utah Medical School. Written informed consent to participate in this study was provided by the participants' legal guardian/next of kin.

## Author Contributions

LE-C, JM, and HK conceptualized and designed the study, drafted the initial manuscript, and reviewed and revised the manuscript. CC made substantial contributions to acquisition of data, critically reviewed, and revised the manuscript for important intellectual content. JM, AC, and RH made substantial contributions to the analysis, interpretation of data, and revised the manuscript for important intellectual content. All authors approved the final manuscript as submitted and agree to be accountable for all aspects of the work.

## Conflict of Interest

The authors declare that the research was conducted in the absence of any commercial or financial relationships that could be construed as a potential conflict of interest.
