# Supplementary material for: As Time Goes by: Understanding Child and Family Factors Shaping Behavioral Outcomes After Traumatic Brain Injury
Source: Front Neurol. 2021 Jul 5;12:687740. doi: 10.3389/fneur.2021.687740 (PMC8287068; doi:10.3389/fneur.2021.687740)
Supplement: Supplementary file 1 [file Table_1.DOCX]

**Supplementary Material**

|  | **1** | **2** | **3** | **4** | **5** | **6** | **7** | **8** | **9** | **10** |
| --- | --- | --- | --- | --- | --- | --- | --- | --- | --- | --- |
| 1. SDQ_ED pre-injury | 1.00 |  |  |  |  |  |  |  |  |  |
| 2. SDQ_ED 3 months | 0.47* | 1.00 |  |  |  |  |  |  |  |  |
| 3. SDQ_ED 12 months | 0.39* | 0.60* | 1.00 |  |  |  |  |  |  |  |
| 4. SDQ_ED 24 months | 0.29* | 0.42* | 0.59* | 1.00 |  |  |  |  |  |  |
| 5. SDQ_ED 36 months | 0.31* | 0.42* | 0.60* | 0.66* | 1.00 |  |  |  |  |  |
| 6. SDQ_CP pre-injury | 0.52* | 0.28* | 0.26* | 0.18* | 0.15* | 1.00 |  |  |  |  |
| 7. SDQ_CP 3 months | 0.31* | 0.39* | 0.34* | 0.23* | 0.25* | 0.65* | 1.00 |  |  |  |
| 8. SDQ_CP 12 months | 0.33* | 0.33* | 0.51* | 0.30* | 0.35* | 0.65* | 0.73* | 1.00 |  |  |
| 9. SDQ_CP 24 months | 0.24* | 0.23* | 0.38* | 0.49* | 0.40* | 0.53* | 0.65* | 0.71* | 1.00 |  |
| 10. SDQ_CP 36 months | 0.10 | 0.16* | 0.30* | 0.29* | 0.42* | 0.45* | 0.66* | 0.68* | 0.68* | 1.00 |

**Table S1. Correlations across SDQ measures and time points**

**Table S2. Summary of Latent Growth Fit Statistics by Model Type**

| Models | SDQ Emotional Difficulties | | | | SDQ Conduct Problems | | | |
| --- | --- | --- | --- | --- | --- | --- | --- | --- |
|  | AIC | aBIC | RMSEA (CI^95^) | CFI | AIC | aBIC | RMSEA (CI^95^) | CFI |
| Linear | 8471.49 | 8482.29 | 0.06 (0.04 – 0.09) | 0.97 | 7608.91 | 7619.70 | 0.05 (0.03 – 0.08) | 0.99 |
| Quadratic | 8467.63 | 8482.75 | 0.07 (0.03 – 0.10) | 0.98 | 7606.83 | 7618.71 | 0.05 (0.02-0.08 | 0.99 |
| Cubic | 8469.01 | 8485.20 | 0.07 (0.04 – 0.11) | 0.98 | - | - | - | - |
| Linear-linear | 8463.97 | 8479.08 | 0.06 (0.02 – 0.09) | 0.99 | 7599.85 | 7614.97 | 0.03 (0.00-0.07) | 1.00 |
| **Quad-linear** | **8454.08** | **8470.27** | **0.00 (0.00- 0.05)** | **1.00** | **7597.51** | **7612.13** | **0.00 (0.00-0.05)** | **1.00** |
